# Supplementary material for: Acceptance and compliance with micronutrient powder (MNP) among children aged 6–23 months in northern Nigeria
Source: PLOS Glob Public Health. 2022 Oct 17;2(10):e0000961. doi: 10.1371/journal.pgph.0000961 (PMC10022258; doi:10.1371/journal.pgph.0000961)
Supplement: S1 Dataset — (ZIP) [file pgph.0000961.s008.zip › S8_Dataset/Spot Checks.zip!/Spot Checks/040715_A_GA_ID_2_SC.pdf]

SPOT CHECK RECORDING FORM

State: __ADAMAWA__LGA: __GANYE__ Community: ____GURUM NUVAN__

Sex of index child: __FEMALE__ Age of index child (in months): __22MONTHS__

Spot Check Information

1. **Date of MNP distribution to household** (circle option below based on LGA name above)**:**
   - **Kebbi:** BirninKebbi&Jega LGA: **June 16**
   - **Kebbi:** Danko-wasagu: **June 17**
   - **Adamawa:**Song, Ganye: **(June 18)**
   - **Adamawa:**Guyuk **June 19**
2. **Date of Spot Check Observation: __8th July, 20I5_____**
3. **Number of sachets remaining** (Ask the caregiver to show you and count the specific number of sachets remaining)**:**

   39

4. **Number of sachets that should remaining one MNP sachet per day** (Analyst only)**:**

   41

5. **Number of sachets remaining / number of sachets should be remaining**(Analyst only)**:**

_39/41__ = __95.1__100%

Probes

Question 1. You have **XX** MNP sachets remaining. Explain how easy or difficult it was to use the MNP product on a daily basis for your child 6 – 23 months? (Circle one answer)

**Very Difficult**	**Difficult**	**(Easy)**	**Very Easy**

Question 2. Describe any specific challenges that you faced that made the MNP difficult to use on a daily basis? No challenges

Question 3. Describe specific aspects of the MNP product that you or your child enjoyed that made it easier to use on a daily basis?

The way it make my child to be eaten food that is why it is easy to use on a daily basis.

Question 4. What questions do you have about the MNP based on your experience using it?
For how long would a child continue to take this mnp before he stop?

Question 5. What specific recommendations do you have that we can do as a program to make the MNP easier to use on a daily basis?

If there is a way in which this kunu can be provided in powdery form and given to us care givers,it will constantly serve as a reminder to administer the MNP on a daily basis.
